# Effect of intraoperative dexmedetomidine infusion on delirium in adult patients following cardiac valve surgery: a protocol of a randomized, double-blinded, and placebo-controlled study

**DOI:** 10.1186/s13063-020-04574-x

**Published:** 2020-07-14

**Authors:** Hong-Bai Wang, Liang Zhang, Zhe Zhang, Su Yuan, Fu-Xia Yan, Qi-Peng Luo

**Affiliations:** 1 Department of Anesthesiology, Fuwai Hospital, Chinese Academy of Medical Sciences and Peking Union Medical College, Beijing, No. 167 North Lishi Road, Xicheng District, Beijing, China; 2 Department of Anesthesiology, Chongqing Traditional Chinese Medicine Hospital, Chongqing, No. 6, 7 Branch Road, Panxi, Jiangbei District, Chongqing, China

**Keywords:** Dexmedetomidine, Cardiac valve surgery, Delirium, Adult

## Abstract

**Background:**

Delirium is an acute status of brain dysfunction that commonly occurs in patients who have undergone cardiac surgery, and increases morbidity and mortality. It is associated with risk factors, such as older age, use of narcotics, cardiopulmonary bypass, and hypothermia. Dexmedetomidine infusion might exert a neuroprotective effect. However, the effect of perioperative administration of dexmedetomidine on the incidence of postoperative delirium (POD) in patients undergoing cardiac or non-cardiac surgery is yet controversial. The present study aimed to reveal the effect of intraoperative dexmedetomidine administration on the incidence of delirium in adult patients following cardiac surgery.

**Methods:**

This single-center, randomized, double-blinded, and placebo-controlled trial consisted of 652 patients randomly divided into two groups: dexmedetomidine and placebo. 0.6 μg/kg dexmedetomidine will be infused 10 min after central vein catheterization, followed by a continuous infusion at a speed of 0.4 μg/kg/h until the end of surgery in the dexmedetomidine group, while normal saline will be administered at the same rate in the placebo group. The primary outcome is the incidence of POD during the first 7 days post-surgery. The secondary outcomes include duration of mechanical ventilation after surgery, duration of stay in the intensive care unit and the hospital after surgery, incidence of hypotension during or after dexmedetomidine infusion, acute kidney injury and sudden arrhythmia during the hospital stay postoperatively, and all-cause mortality in 30 and 90 days after surgery, respectively.

**Discussion:**

This study was approved by the Ethics Committee of the Chinese Academy of Medical Sciences Fuwai Hospital on 6 March 2019 (2019-1180). The results will be disseminated at academic conferences and submitted to peer-reviewed publications. Either positive or negative results will provide guidance for clinical practice.

**Trial registration:**

The Chinese Clinical Trial Registry (http://www.chictr.org.cn) ChiCTR1900022583. Registered on 17 April 2019.

## Strengths and limitations of the study

### Strengths

1. This study includes the largest sample size of all studies with respect to the effect of dexmedetomidine on postoperative delirium in patients undergoing cardiac surgeries.
2. The Chinese Academy of Medical Sciences Fuwai Hospital is a world-class center of cardiac surgery and surgeons and anesthetists with extensive experience in the perioperative management of patients undergoing cardiac surgery.
3. We will select only the patients undergoing cardiac valve surgery to decrease the bias due to a discrepancy in the surgery types.
4. All investigators who will perform the follow-up will have undergone training by a senior psychiatrist about the diagnosis of delirium.
5. The long-term endpoints will be observed.

### Limitations

1. As it is a single-center study, the dissemination of the results may be limited.
2. As some delirium patients may be missed at the follow-up time, a higher incidence of delirium might occur.
3. The Richmond Agitation-Sedation Scale and the Confusion Assessment Method for Intensive Care Unit can produce measurement bias because the two questionnaires are subjective.
4. Lack of interim analysis can lead to an inaccurate sample size, although the sample size in this study is the largest.

## Background

Delirium is a type of brain dysfunction with an acute onset and fluctuating occurrence [1]. According to several clinical studies, the patients have a high prevalence of delirium after cardiac surgery, which varies from 5 to 72% [2, 3]. Several predisposing factors are considered to be associated with the development of postoperative delirium (POD) in patients following cardiac surgery, such as the patients with age ≥ 65 years old [4], narcotics [5], cardiopulmonary bypass (CPB) [6], perioperative poor sleep quality [7], and enhanced postoperative pain sensitivity [8]. POD significantly elevates the incidence of adverse events and 30-day mortality [9–11].

Dexmedetomidine is a medication with a high affinity for α_2_ adrenergic receptor, leading to a decrease in noradrenaline release by activating the α_2_ adrenergic receptors in the central nervous system (CNS), initiating sedation [12]. In addition, dexmedetomidine can be used as a medication for anti-anxiety, improvement of sleep quality, and analgesia [13]. Currently, the drug is widely used in clinical anesthesia and intensive care units (ICU) as an adjuvant sedative.

Interestingly, dexmedetomidine might decrease the incidence of delirium in patients with high risk. A large number of prospective studies about the effect of prophylactic dexmedetomidine on delirium have been published, although most of them included patients undergoing non-cardiac surgeries or were in ICU without surgical procedures [14–21]. Relatively fewer prospective studies have been carried out on heart surgeries, and the majority of these studies adopted the method of perioperative medication [22–24]. Some studies reported intraoperative administration of dexmedetomidine vs. placebo [25, 26]. A prospective study by Shu et al. [25] found that intraoperative infusion of dexmedetomidine significantly decreased the incidence of POD in patients undergoing cardiac surgery as compared to placebo. However, this study only included 60 cases, and the results had poor reliability. Another study with a larger sample size and longer infusion time of dexmedetomidine demonstrated a negative result with respect to the incidence of POD as compared to placebo [23]. Hence, a prospective randomized controlled trial (RCT) with a large-scale sample size is required to further prove the effect of intraoperative dexmedetomidine infusion on POD in patients undergoing cardiac surgery. In this prospective, double-blinded, randomized, placebo-controlled study with a large sample size, we expect to reveal the effect of the intraoperative prophylactic administration of dexmedetomidine at a routine rate on the incidence of POD in patients undergoing cardiac surgery.

## Methods

### Study design

**Fig. 1:**
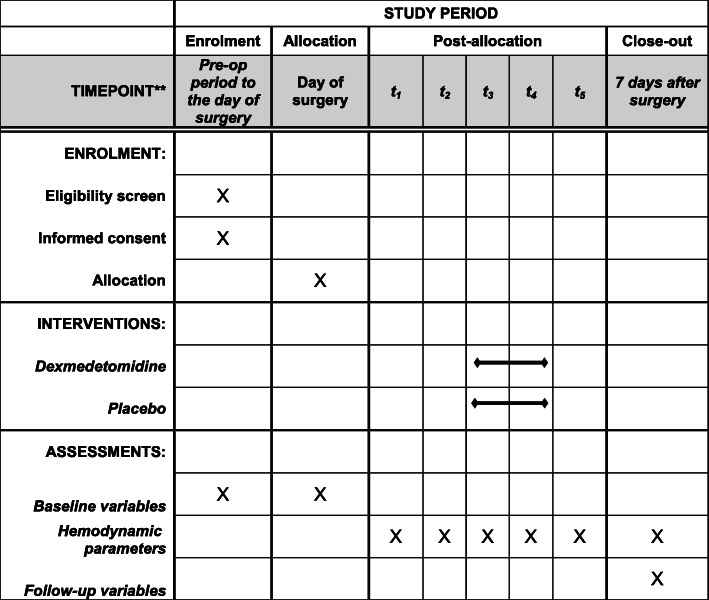
Standard Protocol Items: Recommendations for Interventional Trials (SPIRIT) figure. The schedule of enrollment, interventions, and assessments in the study. t_1_, before anesthetic induction; t_2_, after anesthetic induction; t_3_, after central venous catheterization; t_4_, during surgery; t_5_, after surgery

**Fig. 2:**
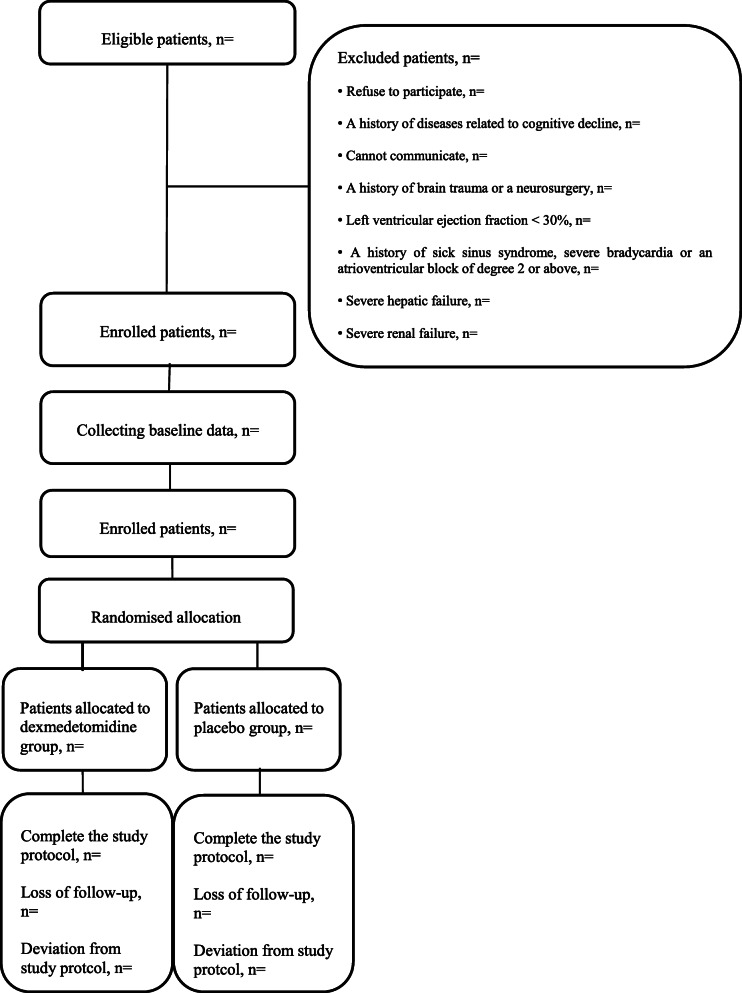
The flow chart of the study

This prospective, double-blinded, randomized, placebo-controlled trial will observe the superiority of dexmedetomidine infusion during cardiac surgery on delirium incidence in the first 7 days after the surgery. The entire progress of trial adheres to the principles of the World Medical Association’s Declaration of Helsinki. An internal Data and Safety Monitoring Board (DSMB) has been constructed to supervise data collection and participants’ safety and will decide on the continuation, modification, or cessation of the trial according to the National Institute of Health (NIH) guidelines. There is no financial and non-financial conflict of interests, as requested by the NIH regulation. The final study version 3.0 was approved by the Ethics Committee of the Chinese Academy of Medical Sciences, Fuwai Hospital, China, on 6 March 2019 (2019-1180). The study was registered in the Chinese Clinical Trial Registry on 17 April 2019 (identification number: ChiCTR1900022583). This protocol includes all components as described in the Standard Protocol Items: Recommendations for Interventional Trials (SPIRIT) checklist (Supplementary file 1) [27]. Figure 1 demonstrates the SPIRIT including the schedule of enrolment, interventions, and assessments of the trial. All eligible patients will be randomly divided into two groups at a ratio of 1:1: dexmedetomidine (DEX) and placebo (PLA). The screening of patients, intraoperative intervention, and postoperative delirium assessment will be performed at the Chinese Academy of Medical Sciences, Fuwai Hospital. The flow chart of this study is shown in Fig. 2.

### Participants

Adult patients (age ≥ 18 years), who are scheduled to undergo open heart cardiac valve surgery on pump under general anesthesia, will be screened for inclusion in this study. Those patients who met either of the following criteria will be excluded: (1) refuse to provide written informed consent; (2) have a history of schizophrenia, epilepsy, Parkinson’s disease, myasthenia gravis, or severe dementia; (3) cannot communicate due to visual, hearing, language, or other barriers; (4) have a history of brain trauma or neurosurgery; (5) have a left ventricular ejection fraction < 30%; (6) have a history of sick sinus syndrome, severe bradycardia (a heart rate < 50 beats/min), or an atrioventricular block of degree ≥ 2; (7) have severe hepatic failure (Child-Pugh Grade C); and (8) have severe renal failure (requirement of renal replacement therapy).

### Patient recruitment and baseline data collection

The investigators will screen the eligible patients the day before the surgery and explain the potential risks and benefits of the study protocol to the patients in person. The patients who do not fulfill any of the exclusion criteria will be eligible for participation in the study. The current study was approved on 26 March 2019. The trial began on 7 May 2019, and over 251 eligible patients have been enrolled by 10 January 2020.

After acquiring written informed consent from the patients, we will collect the following baseline data: demographic data, comorbidity, surgical methods, history of surgeries, and the main results of physical and laboratory examinations. The Pittsburgh Sleep Quality Index [28] and Mini-Mental State Examination [29] will be used to evaluate the preoperative sleep quality and cognition, respectively.

### Randomization, grouping, and blinding

A statistician will complete the generation of random numbers using a computer at a ratio of 1:1 that will then be sealed in envelopes. One of the study personnel, who is not acquainted with the patients before randomization and who will not participate in the anesthesia management or postoperative follow-up of the enrolled patients, will open the envelope according to the order of the random numbers and prepare the study drugs before central venous catheterization.

The investigators who perform the data collection and postoperative follow-up, patients, anesthesiologists, surgeons, and other healthcare team members will be blinded to randomization, study drug preparation, and group allocation during the study period.

An unmasked strategy will be applied immediately if the patients experience any severe adverse event or unexpected worsening of their clinical status. In addition, the research personnel will take effective measures to ensure the safety of the participants. These situations will be documented in the case report forms (CRFs). Then, the unmasked patients will be analyzed only by the intention-to-treat method.

### Interventions, anesthesia, and analgesia

None of the included patients will be permitted to receive premedication as the anticholinergic agent, such as pentylequine or scopolamine. Parameters, such as electrocardiogram, invasive blood pressure, central venous pressure, pulse oxygen saturation, end-tidal carbon dioxide, nasopharyngeal temperature, urine output, and the bispectral index (BIS), will be monitored intraoperatively.

Dexmedetomidine (Jiangsu Hengrui Medicine, Jiangsu, China) (200 μg, 2 mL) and saline will be utilized as the study medicines. A dose of 0.6 μg/kg dexmedetomidine will be diluted in 20 mL saline, and 4 μg/kg will be diluted into 40 mL. The loading dose will be administered as 120 mL/h for 10 min via 20-mL syringes after central venous catheterization, followed by continuous administration at a rate of 4 mL/h (0.4 μg/kg/h) until the end of surgery. The same volume and infusion rate will be applied for patients in the saline group.

The attending anesthetists will attenuate or cease the infusion of the study drug under the following conditions: (1) severe hypotension or bradycardia that does not improve after routine therapy, (2) a worsening or new onset of atrioventricular block of degree ≥ 2 that does not improve after routine treatment, and (3) other conditions. We will record the reason for any protocol deviations in the CRFs and analyze the data from these patients by using the intention-to-treat method but not the per-protocol analysis. We will provide the intervention drugs for free as compensation.

The intravenous medications for anesthesia induction will include midazolam, sufentanil, etomidate, and cisatracurium, and the anesthetics for sustaining anesthesia will include propofol, sufentanil, cisatracurium, and sevoflurane. Additional dexmedetomidine will be infused during surgery in the DEX group according to the study program. The BIS will be used to monitor the depth of anesthesia and maintained between 40 and 60 during surgery. The body temperature will be kept except for mild hypothermia (nasopharyngeal temperature 30–34 °C) during aortic clamping. Mechanical ventilation parameters during off-pump will be set to a tidal volume of 8–10 mL/kg, respiratory rate of 12–14/min, ratio of time of inhalation, and exhalation at 1:2, and the pressure of end-tidal carbon dioxide will be kept between 35 and 45 mmHg. The inhaled gas will be a mixture of air and oxygen, and the fraction of inspired oxygen (FiO_2_) will not exceed 50%. If the pulse oxygen saturation has a continuously low value, and there is no response to treatment involving positive end-exhalation pressure ventilation, a high FiO_2_ will be administered. The hemodynamic stability of the patients will be maintained by adjusting the depth of the anesthesia and applying vasoactive drugs during surgery.

All patients will be transferred to the ICU and shifted back to general cardiac wards after several days. All participants will be equipped with patient-controlled intravenous analgesia (PCIA) pump containing 100 μg sufentanil and 15 mg dezocine in 100 mL saline. The background infusion rate will be 2 mL/h. The pump will deliver an additional 2-mL bolus with a lockout interval of 10 min. Furthermore, intravenous sufentanil or dezocine or oral aminophen oxycodone (oxycodone hydrochloride 5 mg and acetaminophen 325 mg) will be used to control pain after surgery, and the score of the rest numerical rating scale (NRS is a 0–10-point pain scale tool: 0 indicates no pain and 10 indicates worst pain) is over 4 after three consecutive PCIA boluses. Propofol and/or midazolam will primarily be used for sedating the patients after surgery and maintaining the target sedation depth (a Richmond Agitation-Sedation Scale [RASS] score − 2 to 1) [30]. The ICU physicians will perform the treatment of the circulatory system and decide the time of extubation and ICU discharge, according to routine management.

### Outcome assessment

RASS is a questionnaire that evaluates the sedation status of a patient with a score ranging from − 5 to + 4. A score of 0 indicates that the patient is in a calm and awake state. The higher the score, the more severe the patient’s agitation. Conversely, the lower the score, the deeper the patient’s sedation level [31]. The delirium will be assessed using the Confusion Assessment Method for the Intensive Care Unit (CAM-ICU) only when the RASS score is > − 3. The CAM-ICU is an effective tool for assessing delirium, and it contains four parts: (1) sudden variation in mental status and behavior during the past 24 h, (2) inattention, (3) disordered thinking, and (4) an altered level of consciousness. If the patient exhibits (1), (2), (3), or (4), he/she is diagnosed with delirium [32]. Additionally, three subtypes of delirium can be determined using the RASS scores: (1) hypoactive type, RASS score < 0; (2) hyperactive type, RASS score > 0; and (3) mixed type, the hypoactive and hyperactive types occur alternately [33].

### Primary endpoints

The primary endpoint is the prevalence of delirium during the first 7 days after cardiac valve surgery. The investigators, blinded to the preoperative and intraoperative information of the patients, will perform the follow-up of the primary and secondary outcomes. Delirium will be assessed only once on day 1 after surgery within 24 h. From days 2–7 after surgery, the delirium assessment will be performed twice daily (8:00–10:00 and 18:00–20:00) by recording the duration and subtypes of delirium. The occurrence of delirium during actual hospitalization will be considered for patients who are discharged or deceased within 5 days after surgery. In the case of discontinuation of the study drugs during surgery in patients due to side effects or those who undergo emergency surgery again within 7 days after initial surgery, a follow-up will proceed and intention-to-treat analysis will only be used to manage the data.

### Secondary endpoints

The secondary endpoints will include the duration of mechanical ventilation after surgery, the number of days in the ICU and the length of hospital stay after surgery, the incidence of hypotension during and/or after dexmedetomidine infusion, the incidence of acute kidney injury (a ≥ 1.5-fold increment in creatinine from the baseline value) and sudden arrhythmia during the hospital stay after surgery, and the mortality within 30 and 90 days after surgery.

### Safety management

All unexpected events that are unfavorable to the prognosis of the patients throughout the entire medical process will be considered as adverse events. The time, management strategies, and consequences of all adverse events will be recorded in the CRFs. Severe adverse events refer to those that may lead to disability, deformity, or even death, and they will be reported to the Clinical Research Ethics Committee of the Chinese Academy of Medical Sciences, Fuwai Hospital, within 24 h.

#### Delirium

Dexmedetomidine and/or haloperidol will be injected to control the condition. If the patient has recurrent delirium, we will consult a psychiatrist for treatment.

#### Tachycardia

Tachycardia is defined as a heart rate of > 100 beats/min. The management will include adjusting the dose and infusion rate of vasoactive agents, deepening the anesthesia, and administering the β1-adrenoreceptor blocker intravenously.

#### Bradycardia

Bradycardia is defined as a heart rate of < 45 beats/min. The management will involve suspending the surgical operation and adjusting the dose of the vasoactive agent or the infusion rate. If necessary, isoproterenol will be injected intravenously or a pacemaker will be used. The administration of anticholinergic drugs (penehyclidine and scopolamine) will be avoided because of their anticholinergic effect on the CNS.

#### Hypotension

Hypotension is defined as a systolic blood pressure < 80 mmHg or a decrement of > 30% from the preoperative value. The management will include appropriate infusion, adjustment of the anesthesia depth according to BIS, and administration of vasoactive agents.

#### Hypertension

Hypertension is a systolic blood pressure > 160 mmHg or an increment of > 30% from the baseline value. The management will involve adjusting the dose and infusion rate of the vasoactive agents, deepening the anesthesia, and administering a vasodilator intravenously.

#### Arrhythmia

Arrhythmia included frequent atrial or ventricular premature beats, supraventricular or ventricular tachycardia, and the new onset of atrial fibrillation. The treatment strategy will include the suspension of the operation, intravenous infusion of antiarrhythmic drugs, and cardiac cardioversion.

#### Ventricular fibrillation or cardiac arrest

The treatment will include external or intrathoracic heart compression, electrical defibrillation, infusion of epinephrine or other vasoactive agents, intravenous infusion of antiarrhythmic drugs, and an emergency CPB.

### Data management

The original data will be recorded in the CRFs and the EpiData 3.1 software (The EpiData Association, Odense, Denmark) and maintained confidential. Two investigators will be responsible for the collection and entry of the data. Another two independent investigators will contribute to checking the accuracy of the study data. The implementation of the study and collection and entry of the data will be supervised by DSMB. The data management and statistical analysis will be performed by the Biostatistics Department of the Chinese Academy of Medical Sciences, Fuwai Hospital. Because dexmedetomidine is a commonly used medicine in clinical anesthesia, and there have been no reports of serious adverse events, we will not perform an interim analysis.

### Statistical analysis

#### Sample size calculation

PASS 15.0 software (NCSS Corp., Kaysville, UT, USA) is used to calculate the sample size. According to a previous study, the incidence of delirium in patients following cardiac valve surgery was about 13.6% [34]. We estimated a 50% reduction in the incidence of delirium in such patients after an intraoperative dexmedetomidine infusion based on a preliminary experiment by the Chinese Academy of Medical Sciences, Fuwai Hospital. The ratio of the patients’ numbers in the DEX and PLA groups is 1:1. Considering a 5% loss of cases, 652 patients will be required, with a power of 80% and a two-sided *α* level of 0.05.

#### Outcomes analysis

SPSS 22.0 software (IBM Co., Armonk, NY, USA) will be used to analyze the data. The continuous variables that have a normal distribution will be presented as the mean ± standard deviation (SD) and compared using the independent sample *t* test. The continuous data with an abnormal distribution will be presented as the median, interquartile range (IQR), and range and compared using the Mann–Whitney U test. Categorical variables will be expressed as the percentage and compared using Pearson’s chi-square test. The Levene test or the Hodges–Lehmann estimator will be used for estimating the difference (and 95% confidence interval) between the two means or medians. The Kaplan–Meier estimates will be applied for the time to event results. Post hoc subgroup analysis will also be done according to different age gaps of included patients, respectively. A two-sided *p* value < 0.05 will be considered to be a significant difference.

## Discussion

This single-center, prospective, double-blinded, randomized, placebo-controlled trial will observe the effect of dexmedetomidine administration during surgery on delirium in adult patients following cardiac surgery.

A large number of adult patients undergo cardiac surgeries worldwide every year, and coronary artery bypass graft (CABG) and cardiac valve surgery account for the majority of cardiac surgeries [35, 36]. Open heart cardiac surgery is a high-risk factor associated with POD due to the medical interventions, including CPB and hypothermia, during surgery [37]. Another study showed that compared to CABG, the patients undergoing cardiac valve surgery exhibited a high incidence of POD [38]. Therefore, we select patients undergoing cardiac valve surgery as subjects of the current study. Since delirium can occur in all ages of patients undergoing surgery, those included in the current study are not confined to the age group of ≥ 65 years, but to all adults > 18 years of age. The data will be analyzed through post hoc subgroup analysis based on different age groups.

Dexmedetomidine has been widely used as an adjuvant of general anesthesia in patients undergoing cardiac and major non-cardiac surgeries or as a sedative in severe patients in ICU owing to its pharmacological functions of sedation, analgesia, anti-inflammation, and anti-oxidation [39–41]. The dexmedetomidine infusion in this study will begin between the completion of central venous catheterization and the onset of surgery and be maintained until the end of surgery. It will be infused at a routine rate with the loading dose of 0.6 μg/kg for 10 min, followed by 0.4 μg/kg/h to the end of the surgery. Moreover, the potential side effects induced by dexmedetomidine loading dose, including bradycardia, hypotension, and atrioventricular heart-block, under general anesthesia, do not exert significant hemodynamic instability in the patients according to our preliminary test and completed cases. Furthermore, the data about bradycardia and hypotension during or after dexmedetomidine infusion will be recorded as secondary outcomes; we developed effective measures against these effects.

Some intra- and postoperative medical interventions are the main risk factors associated with POD, such as surgery trauma, CPB, anesthetics, hypothermia, infusion, mechanical ventilation, and ICU environment [37, 42]. A previous study included 285 participants from Li et al. [23] and obtained a negative result in POD incidence through a loading dose of 0.6 μg/kg before surgery, followed by a continuous infusion rate of 0.4 μg/kg/h to the end of surgery and 0.1 μg/kg/h after surgery to the end of mechanical ventilation as compared to placebo. However, a prospective study with a sample size of 60 cases from Shu et al. [25] found that intraoperative infusion of dexmedetomidine (a loading dose of 1.0 μg/kg before anesthesia induction, followed by a continuous infusion rate of 0.5 μg/kg/h till the end of surgery) significantly decreased the incidence of POD in patients undergoing cardiac surgery as compared to that of placebo. However, the small-scale sample size made the result of this study lack reliability. Another study displayed that simple postoperative low-dose dexmedetomidine infusion (0.1 μg/kg/h) significantly decreased the POD incidence in patients undergoing non-cardiac surgery as compared to placebo [19]. These findings raised a question: is intraoperative dexmedetomidine infusion required to reduce the POD incidence in patients undergoing cardiac surgery? Therefore, it is necessary to perform two RCTs with large-scale sample size to address this concern based on intra- and postoperative dexmedetomidine administration, respectively.

The sample size is a rough value that varies depending on the results of the references and methods of calculation. According to a prospective screening by Järvelä et al. [34], the incidence of POD was about 13.6% in patients undergoing cardiac valve surgery. However, only a few studies explored the effect of simple intraoperative dexmedetomidine infusion on POD in patients undergoing cardiac surgery. Although the study by Shu et al. showed specific POD incidence after intraoperative dexmedetomidine infusion, it could not be used to estimate the sample size of this study because of its small-scale sample. Therefore, we calculated the sample size of this study through a preliminary test, including 100 cases, and the incidence of delirium was reduced by 50% at our Center through intraoperative dexmedetomidine infusion. Finally, 652 patients were required in this study, which is the largest sample size for investigating the effect of intraoperative dexmedetomidine infusion on POD in patients undergoing cardiac surgery. In addition, an accurate sample size could be obtained by interim analysis, but since dexmedetomidine is a routine clinical medication in our Center, we did not perform interim analysis. This could be a limitation of this study.

Currently, the most commonly used assessment scales for delirium are CAM-ICU and CAM. For patients in ICU, the preference for CAM-ICU scale has become a consensus, while for those in general wards, CAM is preferred to assess delirium [23, 43]. However, the criteria for defining the occurrence of POD are consistent between CAM-ICU and CAM, i.e., the same diagnostic items for delirium include (1) acute onset of mental status changes or a fluctuating course, (2) inattention, (3) disorganization thinking, and (4) an altered level of consciousness. The patients are diagnosed to be delirious if both features 1 and 2 were present in addition to either feature 3 or 4 [32]. Since this study mainly focuses on the occurrence of POD, but not on the extent of delirium, we uniformly used CAM-ICU to assess the occurrence of delirium whether the patients are in ICU or general ward after surgery.

The present study has some strengths. First, it includes the largest sample size of all studies exploring the effect of dexmedetomidine on POD in patients undergoing cardiac surgeries. Thus, this large-scale randomized controlled trial may provide convincing results. Second, our hospital is a word-class center of cardiac surgery. Surgeons and anesthetists have extensive experience in the perioperative management of patients undergoing cardiac surgery. Third, we will select only those patients undergoing cardiac valve surgery to decrease the bias due to a discrepancy in the surgery types. Fourth, all investigators who will perform the follow-up will have received formal guidance about the diagnosis of delirium from a senior psychiatrist; thus, the results of the follow-up will be relatively reliable. Fifth, long-term endpoints will be observed. In this study, the 90-day mortality will be observed, and the long-term endpoints are crucial for evaluating the prognosis of patients.

Nevertheless, the present study had some limitations. First, it is a single-center study, which limits the dissemination of the results. Second, as some delirium patients may be missed at the follow-up time, a high incidence of delirium could occur. Third, although the RASS and CAM-ICU are widely used tools and have high sensitivity and specificity in terms of delirium diagnosis, a measurement bias is inevitable due to the subjective design of the two questionnaires. Fourth, the lack of interim analysis can lead to incorrect sample size, although that in this study is maximal.

### Trial status

The current study (protocol version 3.0) was approved on 26 March 2019. The trial began on 7 May 2019, and over 200 eligible patients have been enrolled until 10 October 2019. We expect to complete the trial in August 2020.

## Supplementary information

**Additional file 1.** SPIRIT 2013 Checklist: Recommended items to address in a clinical trial protocol and related documents.

## Data Availability

All authors will have full access to the final dataset data during the analysis on reasonable request

## Abbreviations

POD: Postoperative delirium
CAM-ICU: The Confusion Assessment Method for the Intensive Care Unit
RASS: Richmond Agitation-Sedation Scale
CABG: Coronary artery bypass graft
DSMB: Data and Safety Monitoring Board
RCT: Randomized controlled trial
CPB: Cardiopulmonary bypass
CNS: Central nervous system
BIS: Bispectral index
CRFs: Case report forms
